# Risk Factors Related to the Death of Admitted COVID-19 Patients: A Buffalo Study

**DOI:** 10.2174/18743064-v17-e230322-2022-21

**Published:** 2023-04-06

**Authors:** Doan Le Minh Hanh, Phan Thai Hao, Do Thi Tuong Oanh, Nguyen Van Tho

**Affiliations:** 1 Department of Internal Medicine, Pham Ngoc Thach University of Medicine, Ho Chi Minh City, Vietnam; 2 University of Medicine and Pharmacy at HCMC, Ho Chi Minh City, Vietnam

**Keywords:** COVID-19, SARS CoV-2, Mortality, Risk factor, BUFFALO, Patients

## Abstract

**Background:**

Coronavirus disease 2019 (COVID-19) may result in a severe acute respiratory syndrome that leads to a worldwide pandemic. Despite the increasing understanding of COVID-19 disease, the mortality rate of hospitalized COVID-19 patients remains high.

**Objective:**

To investigate the risk factors related to the mortality of admitted COVID-19 patients during the peak of the epidemic from August 2021 to October 2021 in Vietnam.

**Methods:**

This is a prospective cohort study performed at the Hospital for Rehabilitation–Professional diseases. The baseline and demographic data, medical history, clinical examination, the laboratory results were recorded for patients admitted to the hospital with confirmed COVID-19. A radiologist and a pulmonologist will read the chest radiographs on admission and calculate the Brixia scores to classify the severity of lung abnormalities. Patients were followed up until beingrecovered or their death. Comparison of clinical and subclinical characteristics between recovery and death groups to find out risk factors related to the death of COVID-19 patients

**Results:**

Among 104 admitted COVID-19 patients, men accounted for 42.3%, average age of 61.7 ± 13.7. The most common symptoms were fever 76.9%, breathlessness 74%, and fatigue 53.8%. The majority (84.6%) of the study population had at least one co-morbidity, including hypertension (53.8%), diabetes (25.9%), gastritis (19.2%), ischemic heart disease (15.4) %), stroke (9.6%) and osteoarthritis (9.6%). The rate of mild and moderate COVID-19 is 13.4%, severe 32.7%, and critical 40.4%. There are 88 inpatients (84.6%) who needed respiratory support. The median hospital stay was 13 days (IQR 10-17.75 days). The rate of intubated patients with mechanical ventilation was 31.7%. The overall mortality rate was 29.8%. Risk factors related to death included Brixia scores > 9, Urea > 7 mmol/L, Ferrtin > 578 ng/ml, Failure to get vaccinated, Age > 60 years, and Low Oxygen SpO2 < 87% (BUFFALO).

**Conclusion:**

The main result of the study is the independent risk factors related to the death of admitted COVID-19 patients including Brixia scores > 9, Urea > 7 mmol/L, Ferrtin > 578 ng/ml, Failure to get vaccinated, Age > 60 years, and Low Oxygen SpO2 < 87% ((BUFFALO) which suggests that these COVID-19 patients should be closely followed up.

## 1 INTRODUCTION

Coronavirus disease 2019 (COVID-19) may lead to the severe acute respiratory syndrome, and results in a major pandemic worldwide. This has been a global health crisis since the flu pandemic in 1918 [1]. There are many risk factors related to severe COVID-19 that were mentioned, such as older age, diabetes, obesity, cardiovascular disease, chronic lung disease, cancer, chronic kidney disease, pregnancy, smoking, organ transplantation, and using immunosuppressive drugs [2]. Although COVID-19 is more and more understood, the mortality rate of admitted COVID-19 patients is obviously high, with several studies providing a mortality rate of up to 61.51% [3]. Therefore, understanding the risk factors of death in relation to COVID-19 patients in each region and each country is always necessary for clinicians to devise treatment strategies, monitor, and prognosticate. During the pandemic outbreak in Ho Chi Minh City of Vietnam, many hospitals received a large amount of COVID-19 patients, including the Hospital for Rehabilitation-Professional diseases. We have conducted this research to understand the risk factors related to death of patients admitted to our hospital during the peak of the epidemic from August 2021 to October 2021, aiming two objectives: (1) Describe clinical, subclinical characteristics and mortality rate of COVID-19 inpatients, (2) Identify mortality risk factors of COVID-19 inpatients.

## 2 MATERIALS AND METHODS

### 2.1 Study Design and Participants

This is a prospective cohort study performed at Hospital for Rehabilitation - Professional diseases from August 2021 to October 2021. Patients who were older than 18 years old, being confirmed COVID-19 according to the diagnosis and treatment guidelines of the Vietnamese Ministry of Health published on July 14, 2021 [1] and consented to participate in the study. Patients who were confused or in a coma and could not give medical history or patients who lost follow-up were excluded from the study.

**Fig. (1):**
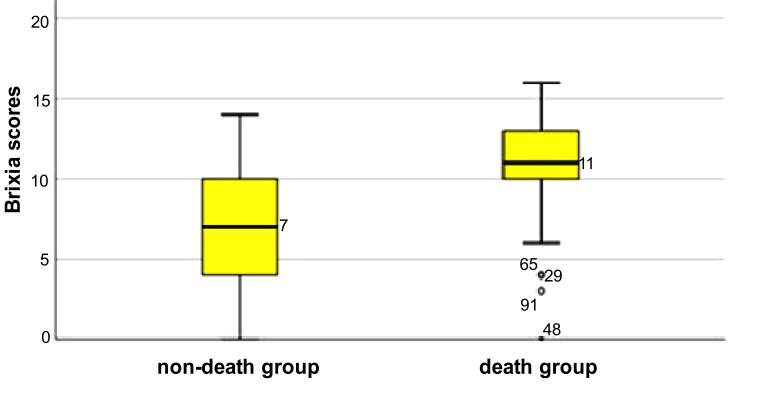
Brixia score in comparision of two groups.

Questionnaires were asked directly to the patients at admission. Baseline characteristics, blood samples and clinical examination were obtained. All patients underwent chest X-ray. A radiologist and a pulmonologist read the chest radiographs on admission and calculated the Brixia scores to classify the severity of lung abnormalities. Patients were followed up until the outcome of recovery or death. Comparison of clinical and subclinical characteristics between recovery and death groups to find out risk factors related to the death of COVID-19 patients (Fig. **1**).

### 2.2 Statistical Methods

Data processing and analysis using SPSS Statistics 20 software and Medcalc 20.1.4 statistical software. Test for normal distribution using the Kolmogorov-Smirnov test. Quantitative variables: data will be presented with mean and standard deviation (if normally distributed) or median and interquartile range (IQR) (if non-normally distributed). The difference between the two groups was tested using the t-statistic. Qualitative variables: data will be presented as frequencies and percentages. The difference test is based on Chi-squared statistic if the theoretical frequency is greater than 5 and Fisher exact if the theoretical frequency is less than 5. Find risk factors related to mortality outcomes by univariable and multivariable logistic regression analysis with the dependent variable (Y) being the mortality rate. After finding the optimal logistic regression model, we proceed to present the relationship between the independent variables and the outcome variable by the OR and the 95% confidence interval. The relationship is statistically significant when the p-value < 0.05.

### 2.3 Medical Ethics

The study was conducted after being approved by the Ethics Committee of the University of Medicine and Pharmacy in Ho Chi Minh City. (Decision No. 2196/QD-ĐHYD). The study did not harm the health of the patients and had voluntary participation from the patients. All personal information is kept confidential. The study did not affect the diagnosis, treatment, and follow-up strategies of patients. The structure and content of the questionnaire did not violate medical ethics or any social norms.

## 3 RESULTS

### 3.1 Clinical Characteristics of the Study Population

During the period from August 2021 to October 2021, we recruited 108 patients who met the inclusion criteria. There were 4 patients excluded from the study due to being transferred to the other hospital according to their family's wishes and losing follow-up. Therefore, 104 participants were included in the final analysis. Men accounted for 42.3%. The average age was 61.7 ± 13.7; the youngest was 25, and the oldest was 92 years old. The mean age of the death group was 65.9 ± 11.2 years and the non-death group was 59.9 ± 14.4 years. The mean BMI of the study population was 23.8 ± 3.4 kg/m2. Most patients were admitted to the hospital on a median day of illness of 7, IQR of 4 to 8 days.

Chief complaints in the study were dyspnea (70.1%), fever (6.7%), lethargy (6.7%), dry cough (4.8%), and cough with phlegm (4.8%). The most common symptoms were fever (76.9%), shortness of breath (74%), and fatigue (53.8%). Less common were anorexia (36.3%), cough with phlegm (31.7%), and dry cough (30.8%). Loss of taste, smell and sneezing, runny nose, and stuffy nose accounted for 20.2% equally. The least common clinical symptoms are abdominal pain, urticaria, maculopapular, and erythematous papules (1%).

There were 56 patients, accounting for 53.8% of the study population, who received one dose of SARS-CoV-2 vaccine, and 3 of these received the second dose. In patients who received the first dose of vaccine, 27 (26%) cases were vaccinated with Astra Zeneca's vaccine, 12 (11.5%) cases were vaccinated with Moderna's vaccine, 17 (16.3%) cases were vaccinated with Sinopharm's vaccine, none of the patients at the time of the study received Pfizer’s vaccine. The three patients who received the 2nd injection were vaccine of Astra Zeneca (1 case), Pfizer (1 case), and Moderna (1 case). The average time from vaccination to disease onset was 23.9 ± 16.8 days, the shortest was 1 day, and the longest was 79 days. The mortality rate in the vaccinated group was 23.2%, and in the non-vaccinated group was 37.5%. In addition, the majority (84.6%) of patients had at least 1 underlying disease. The median number of comorbidities was 1, IQR 1 - 2 diseases. The most common condition was hypertension (53.8%), followed by diabetes (25.9%), gastritis (19.2%), ischemic heart disease (15.4) %), stroke (9.6%), osteoarthritis (9.6%). The median number of comorbidities in the death group was two diseases and in the non-death group was one disease.

Most of the patients in the study had a tachycardia, an average of 99.8 ± 13.9; the respiratory rate increased by a median of 26 breaths/minute (IQR 22-30); SpO2 decreased with a median of 87.5% (IQR 77%-92.5%). The death group compared with the non-death group had a higher respiratory rate (32 versus 24 breaths/minute) and a lower SpO2 (78% versus 91%).

### 3.2 Lab Test Results

White blood cells in most of the study population increased, in which Neutrophil accounted for the majority. The average lymphocyte count was within normal limits, and the percentage of lymphocytes was slightly decreased. The average number of red blood cells, hemoglobin and hematocrit were within normal limits. The mean platelet count in the study was within the normal range; however, the mean platelet count in the COVID-19 death group was lower than in the recovered group (Table **1**).

Blood biochemical tests such as glucose fasting, urea, creatinine, eGFR, sodium, and ALT have average values ​​within normal limits, only AST slightly increased. The mean of Ferrtin, CRP, and D Dimer increased. The cut-off cycle threshold of the real-time RT-PCR cycle was 23.4 ± 4.8 The median sodium concentration in the mortality group was lower than in the recovered group. Our study also recorded an increase in AST levels in the death group compared to the recovered group (Table **2**)

The mean D-Dimer, Ferritin increased in the mortality group in comparison to the recovered group. The mean CRP was high at 62.7 mg/L and the death group was higher than the non-death group (Table **2**).

In terms of electrocardiographic characteristics, our study recorded more than half of the patients with at least one abnormality on the ECG, in which arrhythmias and T-wave abnormalities were the most common, accounting for 15.7% and 14.7% respectively.

Most of the patients in the study had disorders on chest X-ray in the forms of interstitial infiltration and nodular meshwork 71.2%, alveolar opacity 61.5%, consolidation 31.7%, and ground glass opacity 23.1%. Most of the patients had lesions on both sides of the lungs 97.1%, mainly in the outer third (>90%). Most (98%) have diffused and heterogeneous opacity. More than 90% of cases are concentrated in the lower third of both lungs.

The median brixia radiographic score was 8, interquartile range was 5-11. The non-death group had a lower median Brixia score than the death group (chart 1).

### 3.3 Rates of ICU Admission, Mechanical Ventilation, and Mortality of COVID-19 Patients

The rate of mild and moderate COVID-19 was 13.4%, severe at 32.7%, and critical at 40.4%.

73.1% of patients have indications for ICU admission.

88/104 (84.6%) of the patients needed respiratory support, including cannula oxygen, rebreather oxygen mask, high-flow nasal cannula, non-invasive mechanical ventilation, and invasive mechanical ventilation. Twenty-seven patients (30.7%) had to switch to invasive mechanical ventilation during follow-up.

The median hospital stay was 13 days (IQR 10-17.75 days), while the shortest was 3 days and the longest was 36 days.

The rate of intubated patients and mechanical ventilation was 31.7%.

The overall mortality rate was 29.8% (31/104).

### 3.4 Risk Factors Associated with Death

Based on the cut-off points in Table **3**, we conducted univariate and multivariate logistic regression analyses of factors related to the death of COVID-19 patients. The results received 6 risk factors such as age ≥ 60, history of no vaccination for SARS-CoV-2, SpO2 ≤ 87%, Urea > 7 mmol/L, Ferritin > 578 ng/ml, Brixia score > 9, which are independently related factors to mortality of the study population (p<0.05) (Table **4**).

## 4 DISCUSSION

During the pandemic period in 2021, we recruited 104 patients who were admitted to our hospital in emergency conditions. Most of the hospitals in the city were overloaded so COVID-19 patients were often treated at home and hospitalized in severe conditions. Therefore, the average age of the study population was high, approximately 62 years old and the oldest was 92 years old. The mean age of the group of deaths (65.9 ± 11.2 years) was significantly higher (p=0.042) than that of the non-death group (59.9 ± 14.4 years). There was no gender difference between the 2 groups (p > 0.05) while the mean BMI of the non-death group was lower statistical significance than the death group (p=0.027).

The median illness day was 7, IQR of 4 to 8 days. The time of disease onset in the death and non-death groups was not statistically significant (p = 0.615). This time of admission is similar to the record of Davide Ippolito [4] in that most patients are admitted to the hospital after 5 days of disease onset. According to the literature, the time of severe progression requiring hospitalization is usually around 5-10 days [5].

The common chief complaints that led to admission were dyspnea, fever, lethargy, dry cough, and productive cough. Davide Ippolito [4] reported that the main symptoms were fever, cough, and dyspnea. The difference in reasons for hospitalization is the time of admission in our study, which was at the overload period in Ho Chi Minh City; patients were often admitted in severe (32.7%) or critical conditions (40.4%).

The most common symptoms were fever, shortness of breath and fatigue. Less common were anorexia, and cough. The least common clinical symptoms are abdominal pain, urticaria, maculopapular, and erythematous papules. These findings were similar to those of Davide Ippolito [4].

Over half of the study population was vaccinated, and the mortality rate in the vaccinated group was significantly lower than in the non-vaccinated group (p=0.043). The most common condition was hypertension, diabetes, gastritis, and ischemic heart disease. The number of comorbidities in the death group was higher than in the non-death group; this difference was statistically significant (p=0.013). Similarly, Morgan Spencer Gold [6] analyzed 33 studies and recorded 74.4% of deaths with comorbidities and commonly hypertension, diabetes and respiratory disease.

The death group compared with the non-death group had a higher respiratory rate and a lower SpO2; the difference was statistically significant (p<0.001). These results were similar to a study on 601 COVID-19 patients hospitalized at Lombardy hospital in Italy [7], which recorded oxygen saturation and breathing rate to help identify severe patients requiring early non-invasive mechanical ventilation.

There was no difference in the number of white blood cell, neutrophil, and neutrophil percentage between the non-death and death COVID-19 group (p>0.05). Additionally, in the death group, the mean lymphocytes and median percentage of lymphocytes were lower than those of the recovered group (p<0.05). These were similar to the study of Zachary Illg [8].

There were also no significant differences between the two groups who recovered from the disease and died (p>0.05). This result as well as the study of Jianguo Zhang [9] did not find this association.

The mean platelet count in the COVID-19 death group was significantly lower than in the non-death group (p=0.005). The same results were reported by Davood Bashash [3] who analyzed 19 studies, 3383 COVID-19 patients and concluded that decreased platelet count is often associated with severe COVID-19 (Table **1**)

In comparing the 2 groups, we found that urea, creatinine, eGFR, AST, D Dimer, Ferritin, CRP of the mortality group were significantly higher than that of the recovered group (p<0.05). This was like the study of Fen Lan [10]. In contrast, the median sodium concentration in the mortality group was lower than in the recovered group (p=0.008) (Table **2**). Andrea Berni [11] also reported similarly and concluded that hyponatremia was an independent predictor for death on admission (2.7 times higher than normal sodium). Decreased sodium could be correlated with a 14.4% increased risk of death. Elevated liver enzymes were also reported in a retrospective study of 373 COVID-19 patients at 5 hospitals in Madrid [12]. Accordingly, 33.1% of hospitalized patients had an increase in AST. Rise-to-base AST is an independent factor of severe COVID-19 disease

The mean D-Dimer, Ferritin, CRP increased, and the mortality group was significantly higher (p<0.001) in comparison to the recovered group (Table **2**). These results matched with the study’s results of Fei Zhou and Luo [7, 13].

The average value of the cut-off CT of real-time RT-PCR SARS-CoV-2 was no difference between the two groups (p=0.071) (Table **2**). The results of Abdulkarim Abdulrahman’s study2 had the same record that CT had no correlation with COVID-19 disease status.

This finding in ECG disorders was similar to that reported by Brit Long [14]: Sinus tachycardia is the most common abnormality; other abnormalities include supraventricular tachycardia, ventricular arrhythmias, bradycardia, ST segment changes and T wave.

Median brixia radiographic score in the non-death group was significantly lower than the death group (p<0.001). The results of our study are similar to those of Davide Ippolito4. The median Brixia score of our study is lower than that of Agrawal [15] in which the Brixia score (>12 points) is associated with increased mortality from COVID-19. The difference was due to the severity of Agrawal's study population [15] and there was upper double deaths (60.7%) rate than our study (29.8%).

Our study collected patients during the peak period of the pandemic in Ho Chi Minh City; hospitals were overloaded and did not have enough medicine, especially COVID-19 antiviral medicine and lack of medical equipment. Only serious patients were admitted to the hospital, mild and moderate cases were usually recommended to self-monitor at home under the consultants of telemedicine teams. Therefore, the stratification of severity in our study was quite high and most of the patients at admission required respiratory support. The mortality rate in our study is similar to the study of the author Anant Dinesh [16] with the hospitalization rate of 21.26%, the mortality rate in Brooklyn public hospitals is 29.9%, Queens 28.1%, and Manhattan 20.4%.

Based on the cut-off points in Table **3**, we conducted univariate and multivariate logistic regression analyses of factors related to the death of COVID-19 patients. The results received 6 risk factors such as age ≥ 60, history of no vaccination for SARS-CoV-2, SpO2 ≤ 87%, Urea > 7 mmol/L, Ferritin > 578 ng/ml, Brixia score > 9, which were independently related factors to mortality of the study population (p<0.05) (Table **4**). Our results were like that reported by Zelalem G. Dessie [17], who analyzed 42 studies with 423,117 patients with comorbidities, gender, age, smoking status, obesity, and lesions. Acute renal failure and D-dimer are risk factors for admission to the ICU. According to the U.S. Centers for Disease Control (CDC), of 1,228,664 people who completed the first dose of immunization between December 2020 and October 2021, serious outcomes associated with COVID-19 (0.015%) or death (0.0033%) were rare . As such, vaccines are a protective factor against COVID-19, which matches our univariate analysis. Similarly, for respiratory rate variables, SpO2, CRP, Ferritin are the factors associated with COVID-19 mortality. These findings were also reported by Ahmed Mukhtar *et al*. [18] in a retrospective observational study analyzing SpO2 on breathing air within the first 6 hours after hospital admission of COVID-19 patients. 19. The lower SpO2 cut-off point in Ahmed Mukhtar study (78% vs 87%) may be due to the different sample population which was more severe in Ahmed’s research. Huang *et al*. [19] studied 5350 patients who were aggregated from 25 studies with increased CRP (RR 1.84, 95% CI 1.45-2.33, p < 0.00), increased D-dimer (RR 2.93, 95% CI 2.14, 4.01, p < 0.001) was associated with an increased risk of poor outcomes. Besides, according to the results of the retrospective cohort study by Alonso Soto [20], mortality corresponded to elderly, history of surgery, low oxygen saturation, presence of comorbidities, unconsciousness and the number of white blood cells. The model of Alonso Soto [20] has 2 similar factors to our research model: elder and low oxygen saturation are independent predictors of mortality. However, the author's cut-off point for oxygen saturation was lower than ours because the author's study had more severe patients and a higher mortality rate than our study (46% versus 29.8%).

## CONCLUSION

Coronavirus disease 2019 (COVID-19) with diverse clinical symptoms on multi-organs can lead to acute respiratory failure and death. During the outbreak of the pandemic in Vietnam, especially in the year of BUFFALO, most of the COVID-19 patients who were admitted to the hospital were severe and critical indicating ICU admission and needing respiratory support. One-third had to intubate and use mechanical ventilation. Independent risk factors related to the death of COVID-19 included: Brixia score > 9, Urea > 7 mmol/L, Ferritin > 578 ng/ ml, Failure to get vaccinated, Age > 60, Low Oxygen SpO2 < 87% (BUFFALO).

## Figures and Tables

**Table 1:** Comparison of Complete blood count in non-death and death group.

**Complete Blood Count**		**Non-death**	**Death**	**p**
White blood cell 109 / L	Median-IQR	11400 8100-14700	10000 7900-16600	0.555*
		10700 (8000-14900)		
Neutrophil 109 / L	Median-IQR	9200 5900-13300	8300 6400-14400	0.994*
		9050 (6000-13300)		
% Neutrophil	Median-IQR	82,7 75.9-87.0	84,7 81-91.7	0.072*
		83.1 (76.8-88.6)		
Lymphocyte 109 / L	Mean ± SD	1501.4 ± 669.7	1083 ± 1611	0.004**
		1376.7 ± 677.7		
% Lymphocyte	Mean ± SD	12.9 8.9-18.8	10.4 5.5-14.3	0.018*
		11.9 (7.5-17.6)		
Red blood cell 1012 / L	Mean ± SD	4.7 ± 0.7	4.6 ± 0.7	0.860**
		4.7 ± 0.7		
Hb (g/dL)	Mean ± SD	14.4 ± 1.7	14.4 ± 2.2	0.412**
		14.3 ± 1.8		
Hct (%)	Mean ± SD	41.8 ± 5.1	40.6 ± 6.4	0.338**
		41.4 ± 5.5		
Platelet 109 / L	Mean ± SD	242.0 ± 87.4	186.7 ± 93.7	0.005**
		225.5 ± 94.4		

**Note:** IQR: interquartile range; SD: Standard Deviation * Mann-Whitney test ** t test

**Table 2:** Comparison of blood biochemical results and real time RT-PCR between the two groups death and non-death.

**Blood Biochemical Results and Real Time RT-PCR**		**Non-death**	**Death**	**p**
Glucose (mmol/L) n=102	Median-IQR	7.8 (6.4-11.7)	8.3(6.3-12.4)	0.766*
		7.7 (6.2-12.2)		
Ure (mmol/L) n= 103	Median-IQR	4.8(3.5-6.3)	8.7(4.9-11.6)	< 0.001*
		5.3 (4.1-7.5)		
Creatinine (µmol/L) (n= 104)	Median-IQR	72.7 (63.3-87.4)	96.1(76.6-128.6)	< 0.001*
		78.7 (65.5-99.3)		
eGFR MDRD n=104 (mL/phút /1,73 m2)	Mean±SD	79.2 ± 20.6	56.6 ± 24.0	< 0.001**
		72.5 ± 23.9		
Na (mmol/L) n=104	Median-IQR	134 (130-137)	131 (125-134.3)	0.008*
		133 (128-136)		
AST (U/L) n=102	Median-IQR	40.3 (27.2-59.6)	66.7(52.7-101.0)	< 0.001*
		50.6 (32.3-81.5)		
ALT (U/L) n=102	Median-IQR	39 (23.6-68.8)	36.8 (27.7-68.0)	0.904*
		38 (24.6-70.1)		
D-Dimer (ng/ml) n=104	Median-IQR	488 (295-860)	1227 (748-3306)	< 0.001*
		657 (366.8-1405.3)		
Ferritin (ng/ml) n=98	Median-IQR	470 (286-824)	1000 (700-1228)	<0.001*
		576.7 (326.5-1000)		
CRP (mg/L) n=95	Median-IQR	54.3(24.4-105.7)	109.6(52-162.6)	0.004*
		62.7 (32.3-124)		
Threshold cycle RT PCR (n=103)	Mean±SD	24 ± 4.8	22.1 ± 4.5	0.071**
		23.4 ± 4.8		

**Abbreviations:** IQR: interquartile range; SD: Standard Deviation * Mann-Whitney test **t test

eGFR: estimated glomerular filtration rate

MDRD: Modification of Diet in Renal Disease

AST: Aspartate Aminotransferase

ALT: Alanine Aminotransferase

RT-PCR: Real time-Polymerase Chain Reaction

**Table 3:** Sensitivity, specificity, AUC, cut-off points of death-realted risk factors.

** Variables **	** Cut-off point **	** Sensitivity **	** Specificity **	** AUC **	** P value **
Age	≥ 60 years	80.6	46.6	0.625	0.026
Respiratory rate	> 25/minute	83.9	63.0	0.819	<0.001
SpO2	≤ 87%	83.9	64.4	0.800	<0.001
Lymphocyte	≤ 1200/μL	74.2	64.4	0.703	<0.001
Plalete	< 234000/μL	77.4	52.1	0.672	0.004
Ure	> 7 mmol/L	67.7	88.9	0.804	<0.001
Creatinine	> 67.1 μmol/L	96.8	38.4	0.741	<0.001
eGFR	≤ 63.2 ml/ph	61.3%	82.2%	0.767	< 0.001
Na	≤ 126 mmol/L	45.2%	89.0%	0.665	0.006
AST	> 45.4 U/L	83.9%	62.0%	0.730	< 0.001
D-Dimer	> 779 ng/ml	74.2%	71.2%	0.747	< 0.001
Ferritin	> 578 ng/ml	86.2%	66.7%	0.759	< 0.001
CRP	> 39.6 mg/L	89.3%	40.3%	0.691	< 0.001
Điểm Brixia	> 9	77.4%	74%	0.789	< 0.001

**Table 4:** Multivariate logistic regression analyses of factors related to the death of COVID-19 patients.

Variables	OR	Confident Interval 95%	P value
**Age ≥ 60**	5.04	0.01-0.71	**0.025**
**Failure to get vaccinated**	4.34	1.16-114.3	**0.037**
**Cormorbidities**	0.48	0.56-3.31	0.49
**Respiratory rate > 25/minute**	1.76	0.053-6.705	0.67
**SpO2 ≤ 87%**	4.64	0.002-0.750	**0.031**
**Ure > 7 mmol/L**	10.12	0.001-0.174	**0.001**
**CRP > 39.6 mg/dL**	2.48	0.99-1.02	0.116
**Ferritin > 578 ng/ml**	4.45	1.18-79.47	**0.035**
**D-Dimer > 779 ng/ml**	2.45	0.025-1.51	0.12

## Data Availability

The data and supportive information is available within the article.

## LIST OF ABBREVIATIONS

eGFR: = Estimated Glomerular Filtration Rate
MDRD: = Modification of Diet in Renal Disease
AST: = Aspartate Aminotransferase
ALT: = Alanine Aminotransferase
RT-PCR: = Real time-Polymerase Chain Reaction

## STANDARDS OF REPORTING

STROBE guidelines were followed.

## References

[1] (2021). Guidance on diagnosis and treatment of COVID-19 caused by new strain of corona virus (SARS-CoV-2) issued together with decision No. 3416/QD-BYT dated July 14, 2021 of the vietnamese ministry of health.

[2] Axfors C, Janiaud P, Schmitt AM (2021). Association between convalescent plasma treatment and mortality in COVID-19: A collaborative systematic review and meta-analysis of randomized clinical trials.. BMC Infect Dis.

[3] Xu J., Yang X., Yang L., Zou X., Wang Y., Wu Y., Zhou T., Yuan Y., Qi H., Fu S., Liu H., Xia J., Xu Z., Yu Y., Li R., Ouyang Y., Wang R., Ren L., Hu Y., Xu D., Zhao X., Yuan S., Zhang D., Shang Y. (2020). Clinical course and predictors of 60-day mortality in 239 critically ill patients with COVID-19: A multicenter retrospective study from Wuhan, China.. Crit. Care.

[4] Ippolito D., Maino C., Pecorelli A., Allegranza P., Cangiotti C., Capodaglio C., Mariani I., Giandola T., Gandola D., Bianco I., Ragusi M., Franzesi C.T., Corso R., Sironi S. (2020). Chest X-ray features of SARS-CoV-2 in the emergency department: A multicenter experience from northern Italian hospitals.. Respir. Med..

[5] (2021). COVID-19 Treatment Guidelines Panel. Coronavirus Disease 2019 (COVID-19) Treatment Guidelines.. National Institutes of Health.

[6] Gold M.S., Sehayek D., Gabrielli S., Zhang X., McCusker C., Ben-Shoshan M. (2020). COVID-19 and comorbidities: A systematic review and meta-analysis.. Postgrad. Med..

[7] Luo X, Zhou W, Yan X (2020). Prognostic value of C-reactive protein in patients with COVID-19.. Clin Infect Dis.

[8] Illg Z., Muller G., Mueller M., Nippert J., Allen B. (2021). Analysis of absolute lymphocyte count in patients with COVID-19.. Am. J. Emerg. Med..

[9] Zhang J., Hu J., Huang X., Fu S., Ding D., Tao Z. (2022). Association between red blood cell distribution width and COVID-19 severity in delta variant SARS-CoV-2 infection.. Front. Med. (Lausanne).

[10] Lan F., Zhu C., Jin R., Zhou L., Hu Y., Zhao J., Xu S., Xia Y., Li W. (2021). Clinical characteristics of COVID-19 patients with complications: implications for management.. Ther. Adv. Chronic Dis..

[11] Berni A., Malandrino D., Corona G., Maggi M., Parenti G., Fibbi B., Poggesi L., Bartoloni A., Lavorini F., Fanelli A., Scocchera G., Nozzoli C., Peris A., Pieralli F., Pini R., Ungar A., Peri A. (2021). Serum sodium alterations in SARS CoV-2 (COVID-19) infection: impact on patient outcome.. Eur. J. Endocrinol..

[12] Feikin D.R., Higdon M.M., Abu-Raddad L.J., Andrews N., Araos R., Goldberg Y., Groome M.J., Huppert A., O’Brien K.L., Smith P.G., Wilder-Smith A., Zeger S., Deloria Knoll M., Patel M.K. (2022). Duration of effectiveness of vaccines against SARS-CoV-2 infection and COVID-19 disease: results of a systematic review and meta-regression.. Lancet.

[13] Zhou F., Yu T., Du R., Fan G., Liu Y., Liu Z., Xiang J., Wang Y., Song B., Gu X., Guan L., Wei Y., Li H., Wu X., Xu J., Tu S., Zhang Y., Chen H., Cao B. (2020). Clinical course and risk factors for mortality of adult inpatients with COVID-19 in Wuhan, China: A retrospective cohort study.. Lancet.

[14] Long B., Brady W.J., Koyfman A., Gottlieb M. (2020). Cardiovascular complications in COVID-19.. Am. J. Emerg. Med..

[15] Agrawal N., Chougale S.D., Jedge P., Iyer S., Dsouza J. (2021). Brixia chest X-ray scoring system in critically ill patients with COVID-19 pneumonia for determining outcomes.. J. Clin. Diagn. Res..

[16] Dinesh A., Mallick T., Arreglado T.M., Altonen B.L., Engdahl R. (2021). Outcomes of COVID-19 admissions in the New York City public health system and variations by hospitals and boroughs during the initial pandemic response.. Front. Public Health.

[17] Dessie ZG, Zewotir T (2021). Mortality-related risk factors of COVID-19: A systematic review and meta-analysis of 42 studies and 423,117 patients.. BMC Infect Dis.

[18] Mukhtar A., Lotfy A., Hasanin A., El-Hefnawy I., El Adawy A. (2020). Outcome of non-invasive ventilation in COVID-19 critically ill patients: A retrospective observational study.. Anaesth. Crit. Care Pain Med..

[19] Huang I., Pranata R., Lim M.A., Oehadian A., Alisjahbana B. (2020). C-reactive protein, procalcitonin, D-dimer, and ferritin in severe coronavirus disease-2019: A meta-analysis.. Ther. Adv. Respir. Dis..

[20] Soto A., Quiñones-Laveriano D.M., Azañero J., Chumpitaz R., Claros J., Salazar L., Rosales O., Nuñez L., Roca D., Alcantara A. (2022). Mortality and associated risk factors in patients hospitalized due to COVID-19 in a Peruvian reference hospital.. PLoS One.

